# A Molecular Mechanics Energy Partitioning Software for Biomolecular Systems

**DOI:** 10.3390/molecules27175524

**Published:** 2022-08-27

**Authors:** Henrique S. Fernandes, Nuno M. F. S. A. Cerqueira, Sérgio F. Sousa, André Melo

**Affiliations:** 1 Associate Laboratory i4HB, Institute for Health and Bioeconomy, Faculdade de Medicina, Universidade do Porto, 4200-319 Porto, Portugal; 2 UCIBIO–Applied Molecular Biosciences Unit, BioSIM—Departamento de Biomedicina, Faculdade de Medicina, Universidade do Porto, 4200-319 Porto, Portugal; 3 Faculdade de Ciências, Universidade do Porto, Rua do Campo Alegre 1021 1055, 4169-007 Porto, Portugal

**Keywords:** energy partition, molecular mechanics, AMBER, molecular interaction

## Abstract

The partitioning of the molecular mechanics (MM) energy in calculations involving biomolecular systems is important to identify the source of major stabilizing interactions, e.g., in ligand–protein interactions, or to identify residues with considerable contributions in hybrid multiscale calculations, i.e., quantum mechanics/molecular mechanics (QM/MM). Here, we describe Energy Split, a software program to calculate MM energy partitioning considering the AMBER Hamiltonian and parameters. Energy Split includes a graphical interface plugin for VMD to facilitate the selection of atoms and molecules belonging to each part of the system. Energy Split is freely available at or can be easily installed through the VMD Store.

## 1. Introduction

Molecular mechanics (MM) [1] has been widely used in computational studies [2,3,4,5], particularly in large molecular systems in which more accurate methods, e.g., quantum mechanics (QM), cannot be employed. For example, in the study of enzymatic mechanisms [3,6,7,8], MM Hamiltonians are employed in MM minimizations, MD simulations, and QM/MM calculations [9].

During an MD simulation or between steps of a reaction studied with QM/MM [10], the energy of the system varies as a consequence of the rearrangement of the atoms. Such fluctuations can be more or less extensive depending on the sum of all contributions. However, in such large systems, it is difficult to determine which molecules or groups of atoms are responsible for the energy variation. Therefore, a scheme whereby the energy of the system could be split into different parts could help to assess which atoms or molecules contribute the most to this variation. This approach could contribute to the understanding of the contribution of certain amino acid residues to the interaction with a ligand or to identification of which residues contribute to large fluctuations in the MM energy between the minima of a given reaction in a QM/MM study.

In the case of an enzyme with an inhibitor placed at the active site, various active site residues play distinct roles in the interaction with the inhibitor. Therefore, it is necessary to account for the energy contribution of each residue to the interaction, depicting the most important interactions and identifying possible points of stabilization or further stabilization. This information could help to identify superior inhibitor derivatives.

The procedure for studying a catalytic mechanism using a QM/MM approach can also take advantage of an MM energy partitioning technique. In the study of a catalytic mechanism, three structures are typically identified for each step of the mechanism: reactant, transition state (TS), and product. The energy difference between TS and reactant and between product and reactant determines the Gibbs free energy of activation and reaction, respectively. Ideally, the QM portion should contribute the most to these two quantities, as it is the region that includes the atoms involved in the reaction and is treated with the more accurate method. However, in some models, MM portion is responsible for a considerable contribution (more than 10 kcal/mol) to the activation or reaction Gibbs free energies or both. In such cases, the molecular entities involved in the energy variation could be accurately accounted for by including them in the QM region. Because the molecules responsible for the variation are unknown, their inclusion in the QM region is commonly accomplished by trial tests. However, the lack of reasoning for such an approach leads to multiple unfruitful and computationally demanding calculations. The application of an MM energy partitioning technique to the chemical structures of interest could help to identify the species that contribute the most to the energy difference, facilitating the identification of molecules that should be considered in the QM region.

Although capable energy partitioning software programs are currently available [11,12], Energy Split was conceived of and developed to offer a simpler and more compatible way to compare such energies. For example, in the case of a QM/MM calculation, we aim to provide a tool that reproduces the same energy equation in an arithmetic sum of fragments. In order to provide an accessible tool to address the aforementioned issues, Energy Split software was developed using Tcl language (version 8.6) and Tcl threading technology to parallelize the most time-demanding calculations. The proposed tool is particularly useful for macromolecular systems. Therefore, Energy Split was developed considering the AMBER force field, one of the most popular force fields to treat such types of systems.

## 2. Development

### 2.1. Implementation

The MM parameters were applied as implemented in Gaussian [13] software, i.e., for each atom type (i.e., “HP”), the atomic radius (R in Å) (i.e., “1.1000”) and the well depth (ε in kcal/mol) (i.e., 0.0157) are included in the following format:

VDW HP     1.1000     0.0157

These atomic parameters are essential for the calculation of the non-bonded terms: Lennard–Jones and Coulomb potentials. In the case of the Coulomb potential, the atomic charge for each atom is also required. These parameters are directly obtained from the force field, depending on the atom type. However, they can also be derived charges (e.g., RESP) for non-standard molecules.

**Figure 1:**
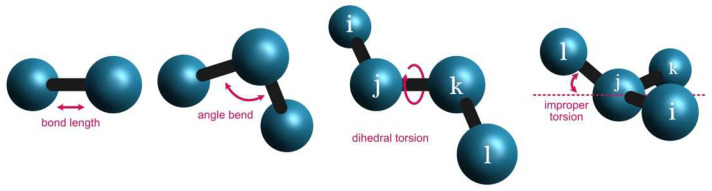
MM bonded terms applied by the AMBER [14] force field.

For each pair of atom types (i.e., “CT” and “HC”) that appears in the system covalently bonded to each other (Figure 1), the bond force constant ($k_{b}$ in kcal/mol/Å^2^) (i.e., “340.00”) and equilibrium bond length ($l_{0}$ in Å) (i.e., “1.0900”) are included:

HrmStr1 CT HC 340.00 1.0900

This approach is similar to the angular bend between sets of three atoms (Figure 1). The trio of atom types (i.e., “C”, “N”, and “H”) is associated with the respective angular force constant ($k_{a}$ in kcal/mol/rad^2^) (i.e., “50.00”) and equilibrium angle value ($\theta_{0}$ in °) (i.e., “120.0001”):

HrmBnd1    C    N    H 50.00 120.0001

In the case of dihedral angles (Figure 1), each set of four sequentially bonded atoms (i.e., “NJ”, “CJ”, “ND”, and “CK”) is included in the parameters section as:

AmbTrs NJ CJ ND CK    0 180 0    0 0.000    4.750    0.000    0.000 1.0

The following four values “0”, “180”, “0”, and “0” correspond to the phase offsets ($A_{i}$ in °). For each phase offset, a magnitude factor ($k_{d}$ in kcal/mol) is also available (i.e., “0.000”, “4.750”, “0.000”, and “0.000”). The last value corresponds to the number of paths (N) (i.e., “1.0”).

Finally, the improper torsions (Figure 1) are calculated based on the following MM parameters:

ImpTrs CJ NJ CD NH     1.1    180.0 2.0

The improper torsion is calculated as the displacement of one atom in relation to a plane defined by three other atoms. Therefore, four atoms are needed to define an improper torsion (i.e., “CJ”, “NJ”, “CD”, and “NH”). The energy contribution of an improper torsion is calculated considering a magnitude factor ($k_{i}$ in kcal/mol) (i.e., “1.1”), a phase offset (A in °) (i.e., “180.0”), and a period (P) (i.e., “2.0”). In the AMBER force field, the central atom for improper torsions is the third atom in the list.

Energy Split was developed to use these parameters, which are available in every Gaussian input file for AMBER calculations, for the respective energy equation:


(1) $$\begin{array}{cc} E_{\mathrm{System}}=\sum_{\mathrm{bonds}}k_{b} & {\left(l-l_{0}\right)}^{2}+\sum_{\mathrm{angles}}k_{a}{\left(\theta -\theta_{0}\right)}^{2}\\ & +\sum_{\mathrm{dihedrals}}\sum_{i=1}^{4}\frac{k_{d}\left(1+\cos\left(i.\phi -A_{i}\right)\right.}{N}\\ & +\sum_{\mathrm{impropers}}\;k_{i}\left(1-\cos\left(P\left(\sigma -A\right)\right)\right)+\sum_{i<j}\left(\frac{A_{ij}}{r_{ij}^{12}}-\frac{B_{ij}}{r_{ij}^{6}}\right)\times S_{LJ}\\ & +\sum_{i<j}\frac{1}{4\pi \epsilon_{0}}\left(\frac{q_{i}q_{j}}{r_{ij}}\right)\times S_{\mathrm{Coulomb}} \end{array}$$

Based on the connectivity data, it is possible to identify which atoms are bonded to each other. Connectivity includes a list of atom indexes that instructs the MM program with respect to how atoms are covalently bonded between to one another. This information is essential to calculate the bonded terms: bonds, angles, dihedrals, and improper torsions. It is also crucial to apply the appropriate scaling factors to the Lennard–Jones ($S_{LJ}$) and Coulomb ($S_{Coulomb}$) potentials.

For each bond, Energy Split calculates the arithmetic distance (l) between the respective ij pair of atoms:

(2) $$l=\sqrt{{\left(x_{j}-x_{i}\right)}^{2}+{\left(y_{j}-y_{i}\right)}^{2}+{\left(z_{j}-z_{i}\right)}^{2}}$$

Then, this distance (l), the equilibrium bond length ($l_{0}$), and the bond force constant ($k_{b}$) are used to calculate the energy contribution of each bond in the system.

The angles are calculated following a similar approach, the first step of which is to calculate the angle between three atoms (ijk). Firstly, the vectors $\vec{ji}$ and $\vec{jk}$ are determined; then, the angle between these vectors is calculated following the dot product expression:

(3) $$\cos\left(\theta \right)=\frac{\;\vec{ji}\cdot \;\vec{jk}}{\;∥\vec{ji}∥\;∥\vec{jk}∥\;}$$

The calculated angle (θ) enters the energy expression with the equilibrium value ($\theta_{0}$) and respective equilibrium value ($k_{a}$).

Dihedral angles are considered any time four atoms (ijkl) are covalently bonded in a sequence. The dihedral angle is, by definition, the angle established between the ijk and jkl planes and calculated by determining the $\vec{ij}$ and $\vec{jk}$ vectors for the ijk plane and the $\vec{kl}$ and $\vec{kj}$ vectors for the jkl plane. The cross product is used to compute the norm vectors for each plane. Finally, the angle between the two norm vectors is calculated using the same approach. Starting with the obtained value, the energy contribution for the dihedral angle is calculated as presented in the respective term in Equation (1).

Improper torsion is calculated by considering all atoms that are exactly bonded to three atoms. Improper torsion occurs when three atoms (ijk) are sequentially connected to each other and atom l is connected to atom j. The concept of improper torsion was developed to provide an energy penalty when atom l exits the ijk plane. The penalization is proportional to the displacement, according to the respective term. Similar to the dihedral angle calculation, two planes are defined: the ijk and ikl planes. Then, the norm vectors are calculated, and the angle between them is calculated as described above.

So far, we have described how the bonded terms are calculated in Energy Split. Most of the operations are implemented using the “math::linearalgebra” module of tcllib (version 1.19), which is available at https://core.tcl-lang.org/tcllib/doc/trunk/embedded/index.md [15].

Finally, the non-bond interactions are calculated considering all atom pairs of the system, as no cutoff was applied. However, this part of the calculation is highly time-demanding and is parallelized through the Thread module of Tcl (https://www.tcl.tk/man/tcl8.6/ThreadCmd/thread.htm version 2.8.7, accessed on 15 January 2022). The calculation of non-bond interactions is split into the number of available threads of the running machine.

The Coulomb term for each pair of atoms is simply calculated as the product of the atomic charges of those atoms ($q_{i}$ and $q_{j}$) divided by the distance between them multiplied by two constants. In an MM calculation, the atomic charges are known and kept fixed for calculation; therefore, only the distance between the atoms needs to be calculated according to Equation (2). Because $\epsilon_{0}$ is the vacuum permittivity, the term $\frac{1}{4\pi \epsilon_{0}}$ is constant and equal to 332.063712827427 kcal·mol^−1^.Å. The Coulomb contribution is also affected by a scaling factor ($S_{Coulomb}$), which depends on how the atoms are connected to each other. In the case of atoms spaced by one or two covalent bonds, the scaling factor is zero because the interaction between those atoms is already accounted for by the bond and angle terms (previously described). With respect to atoms spaced by three covalent bonds, the scaling factor is 1/1.2, following the implementation in Gaussian 09. All other case scenarios are entirely accounted for, so the scaling factor is 1. These values are purely empirical and were set to reproduce accurate calculations and/or experimental data.

The scaling factors are also employed for the calculation of Lennard–Jones potential. In this case, atoms spaced by as many as two covalent bonds are nulled, whereas atoms spaced by three covalent bonds are scaled by 0.5. The scaling for the remaining pair of atoms ($S_{LJ}$) is 1. Similarly to the Coulomb expression, the Lennard–Jones potential depends on the distance between the two atoms involved (ij). Therefore, the distance calculated for the Coulomb term is used to determine the van der Waals contribution. However, the expression for the Lennard–Jones potential requires two additional parameters for each pair of type of atoms: $A_{ij}$ and $B_{ij}$. These two terms are calculated according to the following equations:

(4) $$A_{ij}=\varepsilon_{ij}.D_{ij}^{12}$$

(5) $$B_{ij}=2\varepsilon_{ij}.D_{ij}^{6}$$

In Equations (4) and (5), two variables are required for each pair of atoms. Those variables are calculated based on the atomic radius ($R_{i}$ and $R_{j}$) and well depth ($\varepsilon_{i}$ and $\varepsilon_{j}$) for each atom type. These data are also included in the MM parameters, as mentioned at the beginning of this section. The $e_{ij}$ and $D_{ij}$ terms are calculated according to the combination rule in Equations (6) and (7) (Lorentz-Berthelot rule [16,17]).

(6) $$\varepsilon_{ij}=\sqrt{\varepsilon_{i}.\varepsilon_{j}}$$

(7) $$D_{ij}=R_{i}+R_{j}$$

The MM energy function of AMBER was described in detail, including all the mathematic expressions required for the implementation. The MM paraments stored by Gaussian software are not consistent in terms of units. Therefore, the actual code implementation in Energy Split involves conversion of units.

The implementation of the MM energy function in Energy Split was tested and compared with the results obtained with Gaussian 09 for the same systems. A set of systems was used to compare the computed energies of each individual term: bond, angles, dihedral angles, improper torsions, and van der Waals and Coulomb interactions. Following validation, Energy Split was prepared to split the systems into as many fragments as requested by the user.

**Figure 2:**
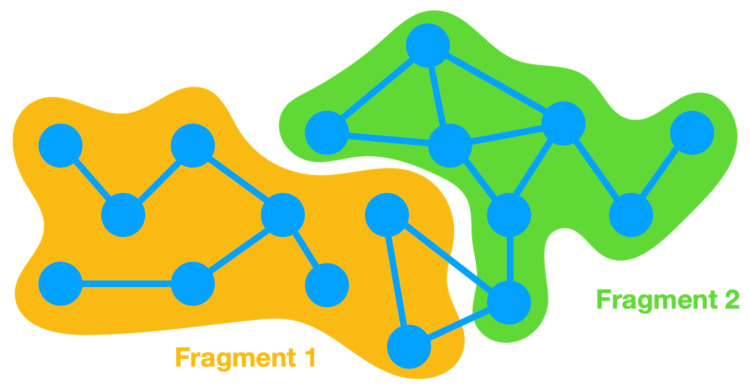
Example of a hypothetical molecular system split into two fragments. The circles and lines represent atoms and covalent bonds, respectively.

In order to clarify the implementation of energy partitioning in Energy Split, an N-atom system is split into two fragments (Figure 2).

Energy partitioning accounts for the various energy terms of each fragment as separate sums. Therefore, in the case of a two-fragment system, two energy sums are included: $E_{frag1}$ and $E_{frag2}$. The energy of fragment 1 ($E_{frag1}$) accounts for the energy of all terms (Equation 1) involving only atoms belonging to fragment 1. The same is applied for fragment 2 and all other possible fragments of the system. A third term is also required to account for the energy of the interactions between atoms that belong to more than one fragment. A covalent bond can span the boundary of two adjacent fragments and is therefore not considered in any of the cases. For such situations, the energy is accounted for in the third term, i.e., the interaction energy between fragments 1 and 2: $E_{int\left(1-2\right)}$. The same is applicable for all other terms wherein the involved atoms belong to different fragments, whether angles, dihedral angles, improper torsions, or van der Waals and Coulomb interactions.

In more complex systems with multiple fragments, the number of interaction terms depends on the interactions between all the atoms involved. Consequently, it is common to find $E_{int\left(1-2\right)}$, $E_{int\left(2-3\right)}$, $E_{int\left(1-3\right)}$, $E_{int\left(2-4\right)}$… The dual fragment interaction term can accommodate all interactions between terms that only involve two atoms (bond term and van der Waals and Coulomb interactions). However, angles can span three fragments, and dihedral angles and improper torsions can involve atoms from four fragments. In such cases, triple (e.g., $E_{int\left(1-2-3\right)}$) and quadruple (e.g., $E_{int\left(1-2-3-4\right)}$) interaction terms are considered.

The total energy of the system is expressed by the sum of all these contributions:

(8) $$E_{system}^{N-frag.}=\overset{N}{\underset{i=1}{\sum }}E_{frag\left(i\right)}+\overset{N}{\underset{\begin{array}{c} i=1\\ i\neq j \end{array}}{\sum }}E_{int\left(i-j\right)}+\overset{N}{\underset{\begin{array}{c} i=1\\ i\neq j\neq k \end{array}}{\sum }}E_{int\left(i-j-k\right)}\;+\overset{N}{\underset{\begin{array}{c} i=1\\ i\neq j\neq k\neq l \end{array}}{\sum }}E_{int\left(i-j-k-l\right)}$$

Energy Split calculates the MM energy of a molecular system in the same manner as standard MM software but offers a straightforward way to split the system into separate fragments. As a result, the energy is calculated in terms of fragments and the interactions between them.

### 2.2. Graphical User Interface (GUI)

**Figure 3:**
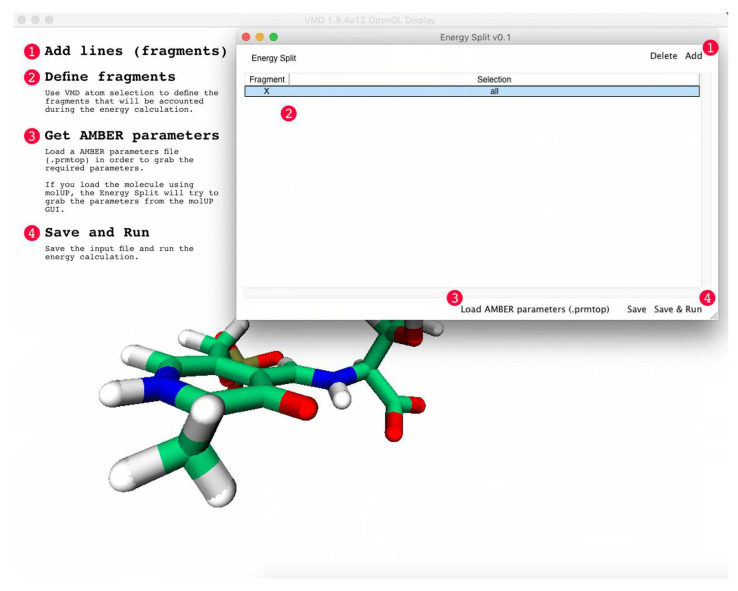
Screen shot of the Energy Split extension for VMD. The labeled numbers and associated text provide instruction on how to use the extension.

A simple GUI was also developed to help users select the fragments for the energy partitioning calculation. The GUI was developed using Tcl/Tk (8.6) language and acts as a VMD [18] extension (Figure 3). The VMD extension was designed to automatically convert the AMBER parameters from a PRMTOP/PARM7 file to the format employed by Gaussian 09 and required for further calculations. Consequently, the user only needs to load the molecular system of interest in VMD using AMBER topology and coordinates. The system can be either an MD simulation or an energy minimization. In the case of multi-frame structures (e.g., an MD simulation), Energy Split automatically selects the current frame in VMD.

**Figure 4:**
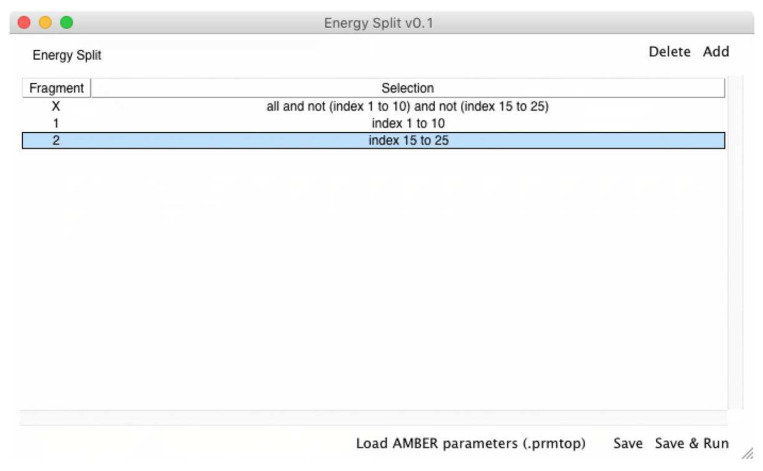
Screen shot of the Energy Split extension for VMD showing a system split into three fragments.

Then, the VMD extension is used to set up the fragments that need to be considered in the energy partitioning calculation. The fragments are defined using VMD atom selections, allowing the user to visually inspect the fragments. The “X” fragment is available by default and corresponds to the remaining molecular system that does not belong to any fragment (Figure 4). However, if one or more fragments, including the “X” fragment, do not contain any atom, Energy Split simply ignores that fragment upon input file generation.

For example, if a system has 30 atoms (indexes 0 to 29) and the user chooses two fragments containing indexes 1 to 10 and 15 to 25, respectively, fragment “X” will contain the remaining indexes, namely indexes 0, 11 to 14, and 26 to 29. (Figure 4)

Then, the AMBER parameters file (PRMTOP/PARM7 file) needs to be loaded into the Energy Split extension window. This process ensures that the MM parameters are evaluated and prepared for the subsequent calculation by Energy Split. The PRMTOP/PARM7 file used in this step should be the same as that used to load the molecular system in VMD.

Finally, the user can choose to save the Energy Split input file to start the calculation in VMD or on another machine or simply click “Save & Run” to save the input file and immediately start the calculation.

The Energy Split extension for VMD was also developed to work seamlessly with the molUP [19] plugin. Therefore, users can load Gaussian input/output files of QM/MM or MM calculations using molUP and define fragments using the Energy Split extension. In this scenario, there is no need to load AMBER parameters from a PRMTOP file, as the MM parameters are automatically imported from molUP.

### 2.3. Calculation Output

An Energy Split calculation can be started directly from the Energy Split extension for VMD by clicking on “Save & Run”. Otherwise, users can save the Energy Split input file and start the calculation in the terminal by running the following command:

tclsh *energySplitPath*/energySplitCalculation.tcl <inputfile>.tcl

Energy Split automatically detects the number of available CPU cores to parallelize the most time-demanding tasks. Once the calculation is finished, the output of the calculation has the following structure:


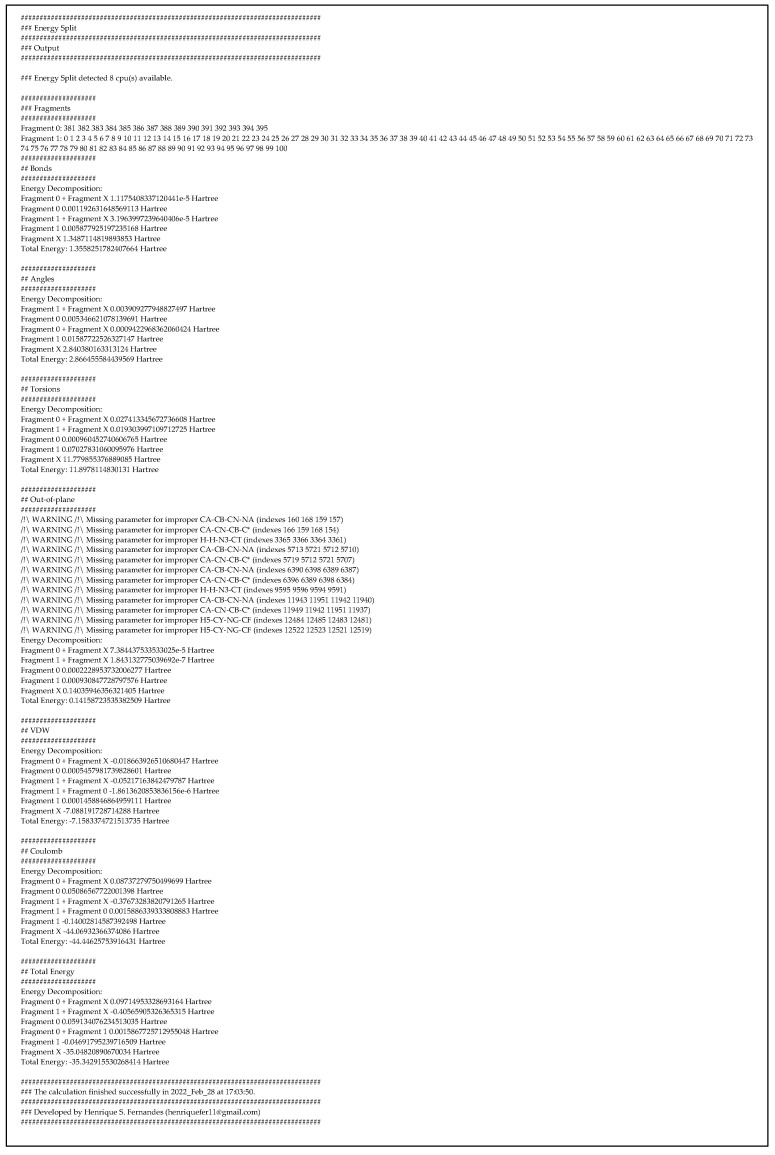


The output provides information about how many CPU cores were detected and used during the calculation. Then, a “Fragments” section lists all the fragments considered in the calculation, with each fragment is followed by a list of atoms included in that particular fragment (atom indexes). In the above example, two fragments were defined by the user (Fragments 0 and 1). Therefore, the calculation was accomplished considering three fragments: fragments 0, 1, and X.

Then, four sections are presented corresponding to the energy calculation for all bond terms: bonds (Bonds), angles (Angles), dihedral angles (Torsions), and improper torsions (Out-of-Plane). The improper torsions are presented with warnings about missing parameters as a consequence of how improper torsions are evaluated in Energy Split. Because Energy Split considers all atoms bonded to exactly three other atoms as potentially improper torsions, some of those calculations are not accomplished due to a lack of MM parameters. The warning is displayed only to advise users of such a situation, suggesting that such atoms should be carefully checked.

After these four sections, the energy for van der Walls (VDW) and Coulomb (Coulomb) interactions are displayed. Finally, the total energy of the system (Total Energy) is presented.

For all sections, the energy is presented following the energy partitioning described by Equation (1). The energy of each fragment is presented as “Fragment *N*”, where *N* can be “0”, “1”, or “X”. In addition, the interaction energy, which corresponds to energy terms that involve atoms from different fragments, are represented as “Fragment *N* + Fragment *M*”, where *N* and *M* can be “0”, “1”, or “X”, and *N* ≠ *M*.

## 3. Example Case: High MM Reaction Energy in QM/MM

An example of the application of Energy Split to identify the source of large energy contributions from the MM portion in a QM/MM study will be provided in this section.

**Figure 5:**
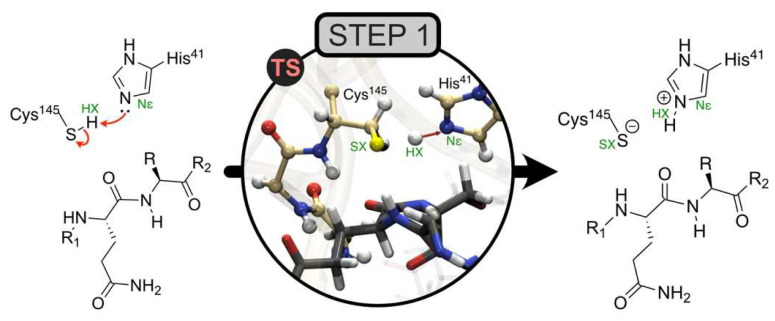
Step 1 of the catalytic mechanism of the SARS-CoV-2 main protease. Transition state image of the active site studied by QM/MM methods in [20]. Adapted with permission from Ref. [20]. 2022, Springer Nature.

This example involves the first step of the reaction mechanism catalyzed by the main protease of SARS-CoV-2 (M^pro^) [20]. M^pro^ catalyzes a set of key reactions essential in the viral replication cycle of the SARS-CoV-2 virus. In the first step, His41 activates the nucleophilic character of the Cys145 residue, allowing for the subsequent nucleophilic attack on the substrate. Therefore, step 1 consists of SG proton transfer from Cys145 to His41 (Figure 5).

For our first hypothesis with respect to this mechanism, a small QM region was used, and the estimated reaction energy of 8.7 kcal/mol had an MM contribution of 6.5 kcal/mol. This result is evidence that a major change occurred in the MM region during the reaction and that the associated molecular species should be included in the QM region for a better description.

**Figure 6:**
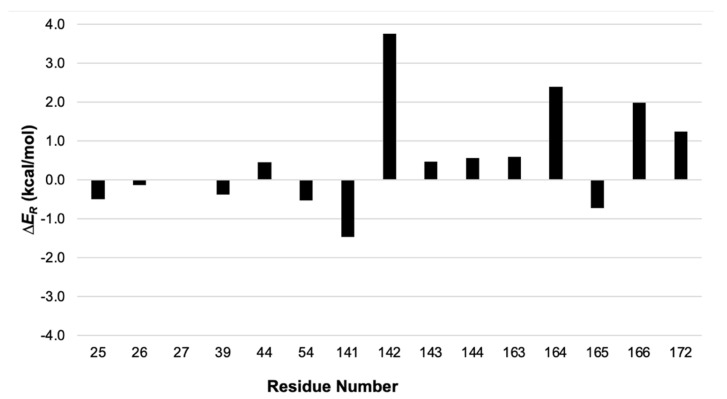
Screen shot of the Energy Split extension for VMD showing a system split into three fragments.

Energy Split can provide valuable help in such situations, highlighting the residues that contribute the most to the MM term of the reaction energy. Therefore, we listed all protein residues within 3 Å from the active site that were in the MM region (residue numbers 25–27, 39, 44, 54, 141–144, 163–166, and 172). Then, Energy Split was used to calculate the MM energy partition for each residue in the reactant and product structures. Finally, the energy differences were calculated for each residue and plotted in Figure 6.

Energy Split allows for a quick depiction of which residues contribute most to the MM term for the reaction energy. These insights could be used to select the residues to include in a larger QM region for further study of the catalytic mechanism [20].

Regarding performance, Energy Split was able to compute one fragment in 5 min and 28 s for a system with 15,216 atoms. This calculation was performed on an AMD Ryzen 5 3600 (12 threads). In a scenario in which the system was split into 17 fragments, the same calculation took 7 min and 57 s.

## 4. Conclusions

Energy Split is a software program for energy partitioning using the AMBER energy function. The main goal is to provide a quick and easy-to-use tool to evaluate the contribution of various parts of a molecular system to its total energy.

Currently, the software is implemented in Tcl with parallelization for the most time-consuming part of the calculation, which corresponds to the energy term for non-bond interactions.

As mentioned, we have identified two potential applications this type of analysis, in particular, the identification of molecular species that have a strong contribution to the activation and/or reaction Gibbs free energies in QM/MM calculation, or the identification of the most important interaction between two molecules during an MD simulation.

The energy partitioning of a single structure does not have a physical meaning; the importance of this strategy arises when multiple related structures are compared in terms of their partial contributions. For instance, energy partitioning can be useful to identify the amino acid residues that account the most for a particular interaction during an MD simulation. Moreover, it could be used to identify possible repulsive interactions that compromise the efficacy of a drug.

The current version of Energy Split and the associated VMD extension are available for free at https://github.com/BioSIM-Research-Group/energySplit and in the VMD Store (https://biosim.pt/software).

## Data Availability

The software described herein is freely available in a public repository at https://github.com/BioSIM-Research-Group/energySplit (added to the repository on 4 February 2019) and can be easily installed through the VMD store (https://biosim.pt/software).

## Sample Availability

No new samples were generated in this study.
